# Systematic mapping of two component response regulators to gene targets in a model sulfate reducing bacterium

**DOI:** 10.1186/gb-2011-12-10-r99

**Published:** 2011-10-12

**Authors:** Lara Rajeev, Eric G Luning, Paramvir S Dehal, Morgan N Price, Adam P Arkin, Aindrila Mukhopadhyay

**Affiliations:** 1 Physical Biosciences Division, Lawrence Berkeley National Laboratory, 1 Cyclotron Road, Berkeley, CA 94720, USA

## Abstract

**Background:**

Two component regulatory systems are the primary form of signal transduction in bacteria. Although genomic binding sites have been determined for several eukaryotic and bacterial transcription factors, comprehensive identification of gene targets of two component response regulators remains challenging due to the lack of knowledge of the signals required for their activation. We focused our study on *Desulfovibrio vulgaris *Hildenborough, a sulfate reducing bacterium that encodes unusually diverse and largely uncharacterized two component signal transduction systems.

**Results:**

We report the first systematic mapping of the genes regulated by all transcriptionally acting response regulators in a single bacterium. Our results enabled functional predictions for several response regulators and include key processes of carbon, nitrogen and energy metabolism, cell motility and biofilm formation, and responses to stresses such as nitrite, low potassium and phosphate starvation. Our study also led to the prediction of new genes and regulatory networks, which found corroboration in a compendium of transcriptome data available for *D. vulgaris*. For several regulators we predicted and experimentally verified the binding site motifs, most of which were discovered as part of this study.

**Conclusions:**

The gene targets identified for the response regulators allowed strong functional predictions to be made for the corresponding two component systems. By tracking the *D. vulgaris *regulators and their motifs outside the *Desulfovibrio *spp. we provide testable hypotheses regarding the functions of orthologous regulators in other organisms. The *in vitro *array based method optimized here is generally applicable for the study of such systems in all organisms.

## Background

Signal transduction to sense and respond to environmental and intracellular changes governs key cellular regulatory functions. In bacteria, two component systems, composed typically of a sensor histidine kinase (HK) and a response regulator (RR), are the primary and best-studied mechanisms for perceiving such changes and controlling downstream responses [1-3]. The regulatory network of an organism is often a reflection of the environments in which it can survive and the signal transduction systems in microbes have been correlated to their sensory IQs [2]. *Desulfovibrio vulgaris *Hildenborough, an anaerobic sulfate reducing bacterium, occupies a variety of ecological niches and encodes a strikingly large number of these systems with unusual diversity attributed to lineage-specific expansion of existing gene families [4]. Studied since the 1940s, *D. vulgaris *Hildenborough has come to serve as a model system to evaluate dissimilatory sulfate reduction and hydrogen cycling [5]. However, the function of none of its two component systems, encoded by 72 RRs and 64 HKs, have been characterized to date.

The distribution of RRs in *D. vulgaris *Hildenborough is considerably different from in other microbes. Of the 72 RRs in *D. vulgaris *Hildenborough, 29 have a DNA binding domain (DBD), indicating function via gene regulation. Twenty-two of these fall into the NtrC family of σ54-dependent RRs. σ54-dependent response regulators in bacteria typically make up approximately 9% of the total RRs in most organisms [2] but in *D. vulgaris *Hildenborough this group constitutes 30% of the total RRs, and 75% of the ones with DBDs. On the other hand, the OmpR family, which typically constitutes the most abundant class of RRs in bacteria, has only two representatives in *D. vulgaris *Hildenborough. The remaining five RRs fall into the LytR and NarL families (Table S1 in Additional file 1). With the exception of DVU1083, which is an ortholog of the *Escherichia coli *PhoB [6], none of the RRs have any characterized orthologs. The targets of these 29 RRs represent the transcriptional portion of the two component regulatory network of this organism and to date remains almost entirely undetermined.

With the exception of a few model organisms such as *E. coli *[7,8], *Caulobacter crescentus *[9], and *Bacillus subtilis *[10,11], genes regulated by these systems remain largely unmapped in most organisms. Even in model bacteria, systematic approaches to delineate gene targets and regulatory networks controlled by two component systems are rare and the available knowledge of their networks represents information compiled from a large body of literature, *in silico *efforts [8,12], or by indirect inference of targets based on transcriptomics analysis [10]. While mapping of binding sites via ChIP-on-chip assays is now done routinely for transcription factors, it is effective for two component RRs only if the activating signal or conditions are known. As a result, even in *E. coli *and *B. subtilis *the function and targets of some two-component systems remain unmapped.

Here we present a systematic experimental determination of the genes regulated by the transcriptionally acting RRs in *D. vulgaris *Hildenborough. We optimized an *in vitro *approach in order to bypass the requirement of using activating conditions that are largely unknown for these two component systems. To our knowledge, this is the first extensive use of an *in vitro *genome-wide method to map bacterial two component system RR binding sites.

## Results and discussion

### Gene targets were determined for 24 *D. vulgaris *Hildenborough RRs

**Figure 1:**
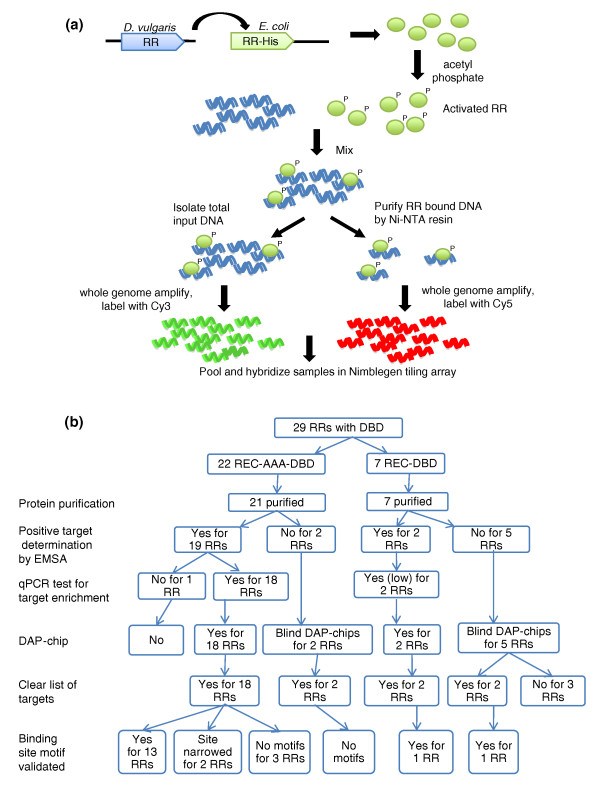
**The DNA-affinity-purified-chip (DAP-chip) method**. **(a) **DAP-chip strategy. The *D. vulgaris *RR gene is cloned into *E. coli *with a carboxy-terminal His-tag. Purified His-tagged protein is phosphorylated with acetyl phosphate, and mixed with sheared *D. vulgaris *genomic DNA. An aliquot of the binding reaction is saved as input DNA, while the rest is subjected to affinity purification using Ni-NTA resin. The input and the RR-bound DNA are whole genome amplified, and labeled with Cy3 and Cy5, respectively. The labeled DNA is pooled together and hybridized to a tiling array, which is then analyzed to determine the gene targets. **(b) **Summary of DAP-chip workflow. The flowchart shows a summary of results at the following steps: protein purification, positive target determination, quantitative PCR (qPCR) test for target enrichment, DAP-chip hybridization, target list determination, and binding site motif validation. AAA, σ54 interaction domain; DBD, DNA binding domain; EMSA, electrophoretic mobility shift assay; NTA, nitrilotriacetic acid; REC, receiver domain; RR: response regulator.

Activation of RRs and downstream effector function via two component systems are highly regulated events *in vivo*. As a result, efforts to identify genes regulated by a given RR *in vivo *necessitate the use of conditions that activate the signal transduction cascade. These signals are known for very few two component systems and are not known for any of the regulators in this study. As such, *in vitro *analyses adapted from ChIP-on-chip-based assays [13-15] provide a reasonable approach. We devised the DNA-Affinity-Purified-chip (DAP-chip) strategy where purified His-tagged RRs are incubated with sheared *D. vulgaris *Hildenborough genomic DNA, and RR-bound DNA is affinity purified using Ni-NTA resin. The enriched DNA fraction and the starting input DNA are whole-genome amplified, labeled with Cy5 and Cy3, respectively, pooled and hybridized to a custom *D. vulgaris *tiling array to determine enriched gene targets (Figure 1a).

**Figure 2:**
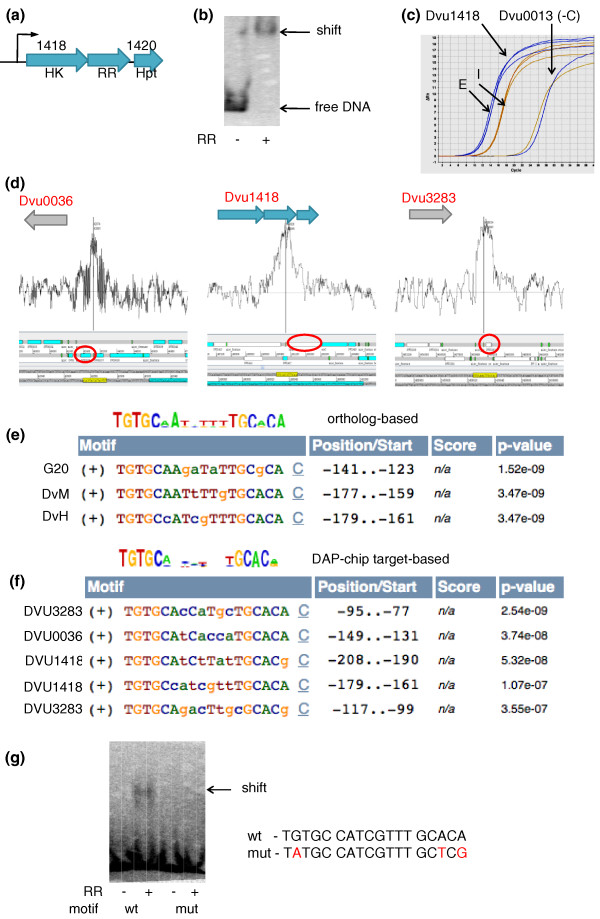
**The steps used to map RRs are illustrated using an example**. **(a) **DVU1419 is a σ54-dependent RR encoded in a three-gene operon. **(b) **RR DVU1419 shifts the upstream region of its own operon. **(c) **Quantitative PCR shows that the upstream region of DVU1418 is enriched in the RR-bound DNA fraction (E) compared to the input DNA (I). As a negative control (-C), quantitative PCR of the upstream region of an unrelated kinase gene, DVU0013, shows no enrichment. **(d) **Artemis plots [52] of the three most highly enriched peaks obtained by DAP-chip. The corresponding gene is highlighted in the red oval. The vertical line corresponds to the location of the binding site motif (highlighted in yellow) within the peak. **(e) **An 18-bp motif was predicted by applying MEME (MicrobesOnline, using PATSER [53]) on the upstream regions of orthologs of DVU1418 in the closely related species *D. vulgaris *Miyazaki (DvM) and *D. desulfuricans *G20. **(f) **A similar motif was found by applying MEME to the upstream regions of the three DAP-chip gene targets. **(g) **Electrophoretic mobility shift assay was used to validate the motif. Changing selected bases within the motif eliminated the shift. DvH, *D. vulgaris *Hildenborough; HK, histidine kinase, Hpt, histidine phosphotransfer domain; mut, modified motif; RR, response regulator; wt, wild-type motif.

**Figure 3:**
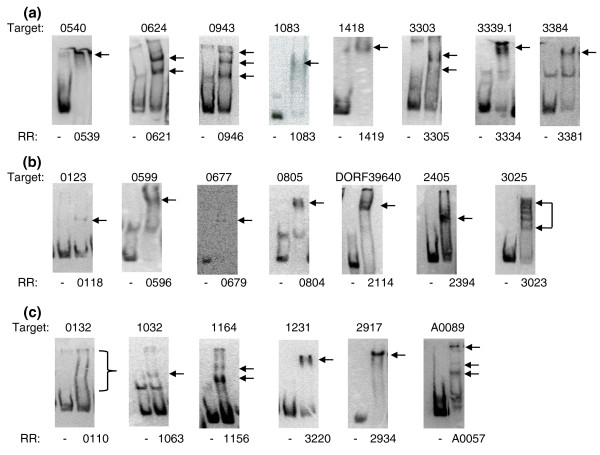
**Determination of a positive target for each RR**. Target numbers indicate the gene DVU# whose upstream region was shifted. RRs are shown by their DVU#. **(a) **RRs whose target was identified upstream of its own gene or operon. **(b) **RRs whose target was identified upstream of adjacent operons. **(c) **RRs whose target was identified based on regulon/promoter predictions (Additional file 2). Arrows indicate shifted DNA. Primers used to amplify the DNA substrates and the sizes of the substrates used are listed in Table S2 in Additional file 1. We used 10 μl of RR in the assay except for RRs DVU621 (5 μl), DVU1083 (4 μl), and DVU3023 (0.5 μl). RR, response regulator.

In our study all RRs being examined present systems with unknown gene targets. To minimize artifacts associated with *in vitro *DNA protein binding assays, we undertook several preliminary experiments to provide the adequate controls to assess false positives and to set the threshold for cut off (outlined in Figure 1b). An example of a completely mapped RR is depicted in Figure 2. We determined one gene target for each of the RRs using gel-shift assays that then served as a positive control. First, the RR was tested for binding to the upstream region of its own gene or operon. If no binding was observed, other candidates were selected for testing based on either their proximity to the RR gene/operon or its regulon predictions (MicrobesOnline [16,17]). For the NtrC family RRs, we also used σ54 promoter predictions to narrow candidates for target genes. Using these rationales, target sequences were successfully found for 21 of the 28 purified RRs (Figure 3; Table S2 in Additional file 1), of which 8 showed binding to sequences upstream of the RR gene/operon itself and 7 had targets in adjacent upstream or downstream operons. For the remaining six RRs, targets were identified as described in Additional file 2.

Parameters for RR binding to the sheared genomic DNA were determined using the gel-shift assays and enrichment of the positive control target in the RR-bound DNA fraction was confirmed using quantitative PCR (qPCR; Table S3 in Additional file 1). Successful enrichment of the positive control and no enrichment of a non-specific negative control (Figure 2c) also serves as a validation of the specificity of binding seen in the gel-shift assays, and increases the confidence in the subsequent DAP-chip data set. The chip-based measurements were then conducted as described in Materials and methods. Nimblescan software was used to analyze the tiling array data and rank enriched gene loci for each RR. The top 20 peaks obtained for each DAP-chip are provided in Table S4 in Additional file 1.

**Figure 4:**
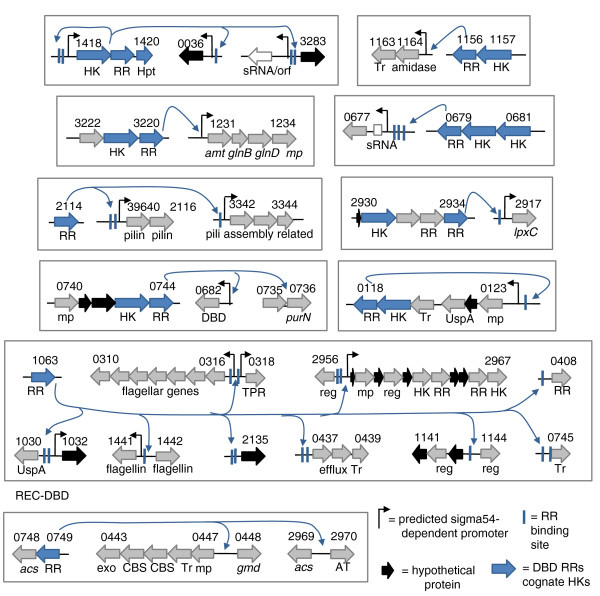
**Single RR regulatory units**. Each box includes one two component system and its targets as determined in this study using DAP-chip. Objects are not drawn to scale. This is a conservative list of the most likely targets, and the complete list of peaks is available in Table S4 in Additional file 1. Where present, predicted σ54-dependent promoters and experimentally validated binding site motifs are indicated. Open boxes indicate missing annotations for sRNAs or ORFs based on the location of the DAP-chip peak and predicted σ54-promoter locations (Figure S1 in Additional file 3). Note that for RR DVU3220, the DAP-chip has not been done, and the target determined using EMSA is shown. AT, acetyltransferase; CBS, cystathionine betasynthase domain; DBD, DNA binding domain; exo, exonuclease; HK, histidine kinase; Hpt, histidine phosphotransfer domain; mp, membrane protein; reg, transcriptional regulator; RR, response regulator; sRNA, small RNA; TPR, tetratricopeptide repeat domain; Tr, transporter; UspA, universal stress family protein domain. Gene names are italicized. For more description see Table S5 in Additional file 1.

**Figure 5:**
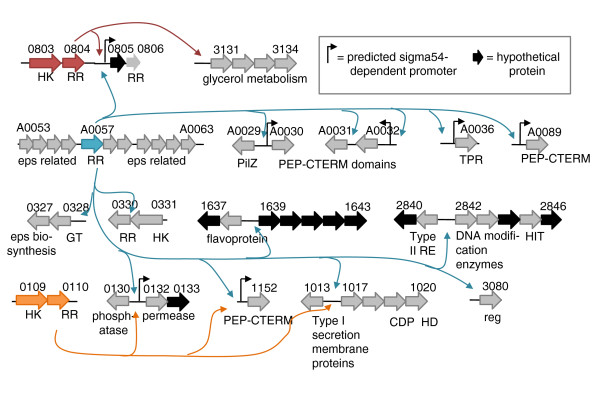
**Regulatory network involving three two component systems**. The RRs DVU0804, DVU0110, and DVUA0057 (each colored differently) have overlapping targets. Objects are not drawn to scale. This is a conservative list of the most likely targets, and the complete list of peaks is available in Table S4 in Additional file 1. Where present, predicted σ54-dependent promoters are indicated. No binding site motifs have been validated for these RRs. CDP, conserved domain protein; eps, exopolysaccharide; GT, glycosyl transferase; HD, HD-GYP domain; HIT, HIT family protein; HK, histidine kinase; PEP-CTERM, Pro-Glu-Pro carboxy-terminal domain; RE, restriction endonuclease; reg, regulator; RR, response regulator; TPR, tetratricopeptide repeat domain. For more gene descriptions see Table S5 in Additional file 1.

**Figure 6:**
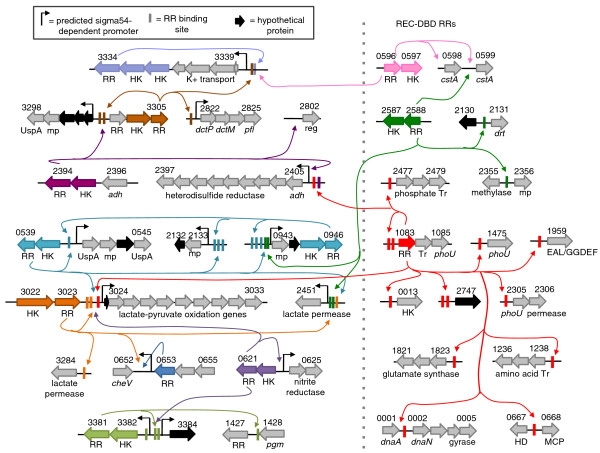
**Regulatory network involving 12 two component systems**. These 12 RRs (each colored differently), including three non-σ54 RRs (shown on the right-hand side), are connected by their overlapping targets. DVU0946 and DVU0539 are paralogs with similar targets and binding sites and are colored the same. Objects are not drawn to scale. This is a conservative list of the most likely targets (for a more comprehensive list of DAP-chip peaks see Table S4 in Additional file 1). Where present, predicted σ54-dependent promoters are indicated. Note that there is a missing gene annotation downstream of DVU0653 (Figure S1 in Additional file 3). Validated binding site motifs (vertical bars) are colored according to the RR gene color. For RR DVU1083, only those targets with a binding site motif are shown in the figure. DBD, DNA binding domain; HK, histidine kinase; MCP, methyl-accepting chemotaxis protein; mp, membrane protein; REC, receiver domain; reg, regulator, RR, response regulator; Tr, transporter; HD, EAL, GGDEF and UspA, protein domain names. Gene names are italicized (Table S5 in Additional file 1).

For all RRs, the DAP-chip assays generated peaks with corresponding low false discovery rate (FDR) scores. Therefore, several criteria (Materials and methods) were used to manually curate the list of most likely targets (Figures 4, 5 and 6; Table S5 in Additional file 1). In most cases the positive target was among the top five candidates on the list (Table S4 in Additional file 1), strengthening the confidence in our data sets. For the seven RRs that had no target positive control determined, DAP-chips were conducted blind (Table S4 in Additional file 1). The blind assays were successful for the two σ54-dependent RRs (DVU0653 and DVU0744) where the targets identified also contained putative σ54-dependent promoters, and for two of the five remaining RRs (DVU0749 and DVU2588). A clear target list could not be identified for RRs DVU2675 or DVUA0137 due to poor overlap in hits from their replicates (Table S4 in Additional file 1). RR DVU2577 had high non-specific binding activity in an electrophoretic mobility shift assay (EMSA). As a result, although the DAP-chip assay for this RR generated a list with some possible targets (Table S4 in Additional file 1), the specificity of these peaks could not be unambiguously determined.

**Figure 7:**
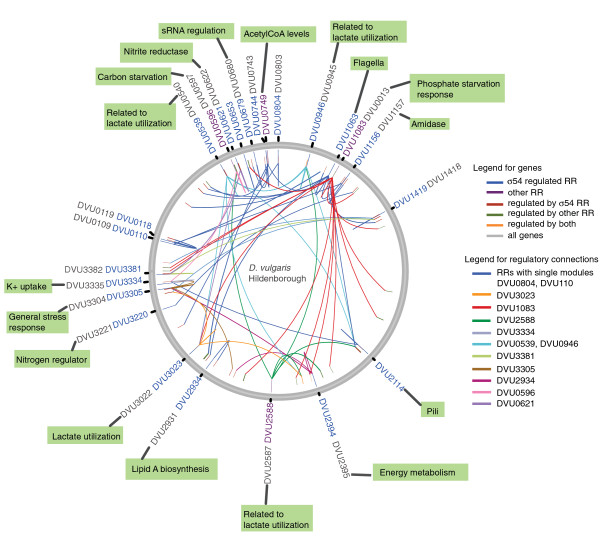
**Regulatory network of *D. vulgaris *Hildenborough. Circular-genome diagram depicting the loci of all RRs, the corresponding mapped genes and predicted functions based on putative annotated functions of target genes**. The figure was generated using a modified Circos script [54]. Genes and connections are as labeled in the figure. Color legends are derived from maps in Figures 4, 5 and 6. Probable functions of RRs as suggested by regulated genes are shown in green boxes. Cognate histidine kinases, where predicted, are shown in gray. Figure does not include pDV1 borne RRs or target genes that map to this plasmid. RR, response regulator; sRNA, small RNA.

Based on our cutoff criteria and EMSA validations, approximately 200 genes (Table S5 in Additional file 1) in 84 operons could be mapped to two component signal control, representing approximately 4% of the ORFs encoded in the *D. vulgaris *Hildenborough genome (Figure 7). The DAP-chip method worked especially well for the σ54-dependent RRs, since σ54 promoter predictions could be used as an additional tool to validate gene targets. The method works best when at least one target is known or can be determined using other methods prior to assaying via DAP-chip. Nevertheless, successful blind DAP-chip measurements are possible for two component systems with no known target, or regulon predictions, as we demonstrated for four RRs.

### Determination of binding site motifs

**Figure 8:**
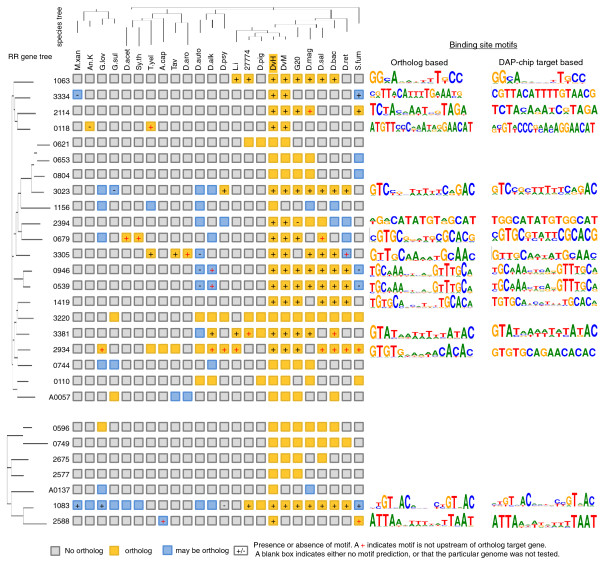
**Phylogenetic profile of the response regulators and their binding site motifs**. The RR gene trees on the left (top half, σ54-dependent RRs; bottom half, the other seven RRs), and the species tree at the top (based on 16S rRNA sequences) were created using FastTree on the MicrobesOnline platform [55]. Numbers on the left indicate RRs by their DVU#. Species abbreviations used: M.xan, *Myxococcus xanthus; *An.K, *Anaeromyxobacter *sp. K; G.lov, *Geobacter lovleyi*; G.sul, *Geobacter sulfurreducens*; D.acet, *Desulfotomaculum acetoxidans*; Sy.th, *Symbiobacterium thermophilum*; T.ye, *Thermodesulfovibrio yellowstonii*; A.cap, *Acidobacterium capsulatus*; Tav, *Thioalkalivibrio *sp. HL-EGBR7; D.aro, *Dechloromonas *aromatica; D.auto, *Desulfobacterium autotrophicum*; D.alk, *Desulfatibacillum alkenivornas*; D.psy, *Desulfotalea psychrophila*; L.i, *Lawsonia intracellularis*; D.pig, *Desulfovibrio piger*; 27774, *Desulfovibrio desulfuricans *27774; DvH, *D. vulgaris *Hildenborough; DvM, *D. vulgaris *Miyazaki; G20, *Desulfovibrio desulfuricans *G20; D.mag, *Desulfovibrio magneticus*; D.sal, *Desulfovibrio salexigens*; D.bac, *Desulfomicrobium baculatum*; D.ret, *Desulfohalobium retbaense*; S.fum, *Syntrophobacter fumaroxidans*. The two other sequenced *D. vulgaris *strains DP4 and RCH1 have an identical set of response regulators as the strain Hildenborough and are not shown in the figure. Orthologs were identified using the ortholog and tree browser functions on MicrobesOnline. Two RRs, DVU1083 and DVU3220, are conserved in all *Desulfovibrio *spp. Experimentally validated binding site motifs for the respective RRs are shown on the right. Motif logos were created using Weblogo [56] by using the sequences shown in Tables S7 (DAP-chip target based) and S9 (ortholog based) in Additional file 1. DAP, DNA affinity purified; RR, response regulator.

**Figure 9:**
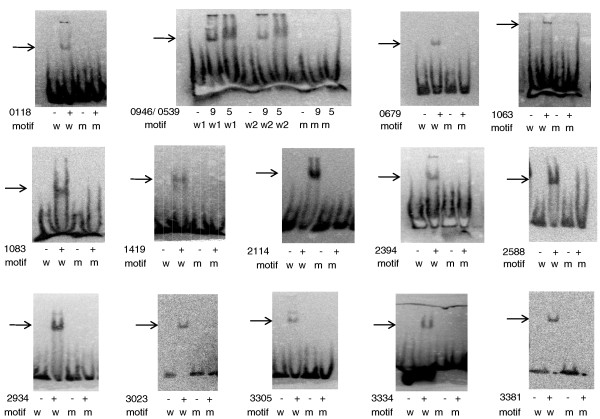
**Validation of predicted binding site motifs using EMSAs**. The sequences tested are in Table S6 in Additional file 1. The RRs are shown by their DVU#. For each RR, a wild type motif (w) and a modified motif (m) were tested. RRs DVU0946 and DVU0539 bind to the same sequence and are shown in one panel. We used 10 μl of the RR prep in the assay, except for RRs DVU3023 (2 μl), DVU3381 (5 μl) and DVU1083 (5 μl). The gel shift observed using the wild-type motif was titrated away using unlabeled wild-type motif but not the modified motif (shown for RR DVU2934 in Figure S5 in Additional file 3). RR, response regulator.

Binding sites for the RRs were determined using two methods. The first method used MEME [18] to find a motif using the upstream regions of the target gene orthologs in the other sequenced *Desulfovibrio *genomes (Figure 8) and was particularly useful for RRs that mapped to a single target locus. The second method used MEME on the upstream regions of the multiple target genes from the DAP-chip results. In most cases, motif finding was more successful using the first method since DAP-chip data were likely to contain sequences that did not correspond to upstream regions or were sticky DNA that did not contain a conserved motif. A reasonable motif prediction was then validated using EMSA on synthesized DNA substrates containing the motif. Where a shift was observed, specificity of the shift was confirmed using synthesized DNA substrates with base pair changes in the predicted conserved sequence (Figure 9). A maximum of three conserved bases within each repeat of the motif was changed as detailed in Table S6 in Additional file 1. For validated motifs that had been predicted based on target orthologs, the DAP-chip peak list was reviewed for other peaks containing the motif. Using the binding sites from the different targets (Table S7 in Additional file 1), the motif was refined to obtain the final binding site motif for the RR (Figure 8).

Our approach proved to be very successful. Binding sites were predicted and confirmed for 15 hitherto uncharacterized RRs (see Figure 8 for the motifs and Figures 4, 5 and 6 for binding site distribution within targets). Experimentally validated motifs further confirm the specificity of the peaks discovered using the DAP-chip method. The majority of the binding sites are palindromic, ranging from 4- to 6-bp inverted repeats separated by 3 to 8 bp in between. In two cases, DVU2394 and DVU1083, the binding site was found to be a direct repeat. Interestingly, RRs DVU0539 and DVU0946, which are paralogs, also recognized the same binding site (Figures 8 and 9).

The confirmed binding motifs were used to assess the general applicability and robustness of this method. First we examined if the binding sequences were present in all the hits for an RR in its DAP-chip data set. Our analyses indicate that for the RRs where a motif was determined, a single motif explained all the targets found in the DAP-chip analysis. The primary exception was DVU1083 (PhoB), where the motif was found upstream of only 14 of the 30 targets. The other exceptions were DVU2394 and DVU0539, where the discovered motif was only present in a subset of the DAP-chip hits (Table S8 in Additional file 1).

A genome-wide scan also revealed that the DAP-chip assay successfully enriched all loci containing the binding site motif for a given RR. An exception to this general observation was the RR DVU1063 (the flagella regulator, see below), for which a sizable number of potential sequences in upstream regions were present that were not enriched as targets in our assay (Table S9 in Additional file 1). Interestingly, many of these are flagella and motility-related genes, suggesting that they are real targets. While it is not clear why these sequences were not enriched in the *in vitro *assay, one possibility is that the quantity of protein or the provided activated state used was insufficient for interaction with all available loci. DNA modification such as via methylation is another source of regulation that would persist in an *in vitro *assay and affect motif recognition. Additional experiments would be required to examine these hypotheses.

### Functional assignments for response regulators

The DAP-chip gene hits enabled predictions for the function of several RRs (Figure 7). Additional genomics data available for *D. vulgaris *Hildenborough from transcriptomics studies under different stresses and conditions (MicrobesOnline) and high-resolution tiling arrays conducted to identify transcribed regions and estimate their level of expression [19] were also used to obtain support for conclusions from our measurements. Using the transcriptomics data, RRs and their target operons were examined for any obvious co-expression patterns while the tiling array data provided support for the expression levels of a gene under routine growth conditions.

#### Lactate utilization is highly regulated

The lactate/pyruvate utilization genes of the operon DVU3025-3033 are regulated by four RRs - DVU3023, DVU0539, DVU0621, and DVU1083 (PhoB). Lactate is the primary electron donor and carbon source for *Desulfovibrio *spp., and the genome of *D. vulgaris *Hildenborough encodes several lactate permeases, lactate dehydrogenases, and pyruvate oxidation genes. Since DVU3025-3033 is so highly regulated, it is likely that it is the primary pathway for lactate utilization. These genes were also observed to be highly expressed in the tiling array data (Figure S1 in Additional file 3) [19]. This operon additionally contains the *ack *gene encoding acetate kinase, which generates acetate from acetyl phosphate, the energy generating step in dissimilatory sulfate reduction. Since acetyl phosphate can act as a small molecule phosphate donor for RRs [20], having multiple regulators for this operon may present a mechanism to modulate acetyl phosphate levels inside the cell.

The targets for the RR DVU3023 are lactate-pyruvate oxidation genes DVU3025-3033, and two lactate permeases (Figure 6), suggesting that the corresponding two component system senses lactate and also plays a role in lactate utilization. Further, the two lactate permeases targeted by DVU3023 are expressed differently. Tiling array data indicate that DVU2451 is expressed highly under normal growing conditions, whereas DVU3284 is not (Figure S1 in Additional file 3) [19]. Gene expression correlations show that the two genes are negatively correlated with each other (Figure S2 in Additional file 3). DVU3023 may be activating DVU2451 expression, while repressing DVU3284.

Additionally the lactate permease gene DVU2451 is also targeted by three RRs -DVU3023, DVU0539 and DVU2588 (Figure 6). These three RRs, along with DVU0946, have common targets that may form a network to fine-tune lactate utilization (see below). Thus, lactate consumption appears to be affected in response to multiple stresses or environmental signals.

#### *D. vulgaris *encodes paralogous RRs with overlapping functions

The RRs DVU0539 and DVU0946 are paralogous and have identical binding sites (Figures 8 and 9), although their corresponding predicted proximal sensor kinases are not paralogous. The two RRs appear to auto-regulate their own expression, and to regulate each other, and the operon DVU2133-2132. DVU0539 additionally regulates DVU3025-3033, and DVU2451 (Figure 6). Further, within the regulated candidates, genes in the operon DVU2133-2132 appear to be paralogs of those in DVU0943-0944. A gene expression correlation map of these candidates shows that DVU0539-0540 transcript levels positively correlate with DVU0542-0545, and DVU2133-2132, but is negatively correlated with DVU0943-0946 and with DVU3025-3033 and DVU2451 (Figure S2 in Additional file 3). Tiling array data support this observation, where during regular growth DVU0943-0946 genes are well expressed, whereas no expression of DVU0539-0545 genes was measured (Figure S1 in Additional file 3) [19]. Since DVU3025-3033 and DVU2451 are lactate utilization genes, it is likely that the functions of the other target genes encoding hypothetical proteins are also tied in to lactate/pyruvate utilization. Taken together these RRs and their target genes present a highly interconnected and feedback controlled regulatory module to control lactate utilization. Despite identical binding motifs used for DNA binding, our findings suggest that DVU0539 and DVU0946 regulate genes differently. Additional experiments, such as the biochemical evaluation of the phosphotransfer from the respective histidine kinases, may shed more light on the mechanism of such regulation.

#### Regulation of lipid A biosynthesis

DVU2934 has a single target, the *lpxC *gene DVU2917 (Figure 4). LpxC is predicted to catalyze the committed step in lipid A biosynthesis. In *E. coli *and *Pseudomonas*, regulation appears to be primarily via control of LpxC protein levels. Excess LpxC in these systems is toxic to the cell, although *lpxC *is an essential gene [21,22]. Tiling array data for *D. vulgaris *[19] suggests that *lpxC *is highly expressed (Figure S1 in Additional file 3). Regulation by DVU2934 may be an additional mechanism to fine-tune its expression. DVU2934 is part of an operon that encodes the histidine kinase DVU2931 and a second RR with a HD-GYP domain (DVU2933), suggesting that cyclic-di-GMP levels may be used to regulate lipid A biosynthesis in *D. vulgaris*.

#### The phosphate starvation response ties in to DNA replication, nitrogen metabolism, and cyclic-di-GMP levels

DVU1083 is annotated as the phosphate starvation response regulator PhoB, and the DAP-chip data support this prediction. Aside from the expected phosphate ABC transporter genes and phosphate transport regulators (PhoU), its targets include DNA polymerase and gyrase genes, amino acid transport genes, glutamate synthase, phosphodiesterase domain (HD/EAL) genes and also some energy metabolism genes, such as the alcohol dehydrogenase and the pyruvate oxidation operon (this also includes the acetate kinase and the phosphotransacetylase (*pta-ack*) genes) (Figure 6). The Pho regulon in other organisms is known to include members that function outside the direct phosphate starvation response, such as in nitrogen assimilation [23], DNA replication [6], and cyclic-di-GMP concentration [24]. The binding site for known PhoB boxes in other bacteria, particularly *E. coli*, consists of two 7-bp direct repeats (consensus CTGTCAT) separated by a 4-bp spacer [25,26]. The *D. vulgaris *PhoB box consensus is a 6-bp repeat with a 4-bp spacer (c/t)GT(n)AC (Figure 8). DVU1083 regulates the DVU2477-2479 operon that encodes the *pstS-C-A *phosphate ABC transporter genes. The *D. vulgaris *genome also has another set of phosphate transporter genes, DVU2667-2663 (*pstS-C-A-B-ATPase*), but interestingly these genes do not have the binding site motif in their upstream regions and were not among the peaks for the PhoB DAP-chip assay.

#### DVU2114 regulates pili assembly

DVU2114 targets the pilin genes DORF39640-DVU2116, and the operon DVU3342-3345 (Figure 4), which encodes pilus assembly genes [27]. Pili are regulated by σ54-dependent RRs in other species such as *Geobacter *[28], *Myxococcus xanthus *[29], and *Pseudomonas *[30]. The type of pilin encoded by DORF39640-DVU2116, and the genes downstream of DVU2116 (DVU2117-2126) appear most similar to the pili assembly machinery (*cpaA-F*, Tad genes) found in *C. crescentus *[31].

#### DVU1063 appears to be a flagella regulator

DVU1063 has a large number of targets for a σ54-dependent regulator, and among these are some flagella-related genes (Figure 4), suggesting its role as a flagella regulator. DVU1063 is homologous to the flagella regulator FlbD in *C. crescentus *[32], and the binding site motif (GGCAxxxxTGCC) resembles that of the *C. crescentus *FlbD (CCC*GG*CAxxxxxTG*CC*GGG), where the italic bases are those that form contacts directly with the RR [32]. Scanning the *C. crescentus *genome with the *D. vulgaris *motif identified several of the FlbD-regulated promoters (not shown). Like FlbD, DVU1063 has an atypical receiver domain that lacks some of the active site residues of the phosphorylation pocket, and it may not require activation by phosphorylation. The regulation of cell motility appears to be complex, since other targets for RR DVU1063 include hypothetical proteins, regulatory proteins, membrane proteins and transporters.

#### Exopolysaccharide and biofilm synthesis is controlled by a pDV1 plasmid encoded regulator

DVUA0057 has been predicted to regulate genes encoding proteins with a PEP-CTERM (Pro-Glu-Pro carboxy-terminal) domain. This domain is predicted to target proteins for export into the exopolysaccharide layer [33]. The RR gene itself is encoded on the native pDV1 plasmid in a ten-gene operon that appears related to exopolysaccharide synthesis. The five predicted PEP-CTERM targets [33] for this RR were enriched in the DAP-chip assay along with several other hits (Figure 5; Table S5 in Additional file 1). The pDV1^- ^*D. vulgaris *strain (lacking the native plasmid) produces three-fold less biofilm than the wild type, and the wild-type biofilm also contains less carbohydrate and more protein filaments [34]. It is possible that DVUA0057 is involved in biofilm formation and that the PEP-CTERM proteins are an integral part of the biofilm. Expression correlations show that most of the PEP-CTERM proteins are positively correlated with each other and DVUA0057 (Figure S3 in Additional file 3). The RRs DVU0804 and DVU0110 have a few overlapping targets with DVUA0057 (Figure 5), and may also be involved in similar functions.

#### A three-component system for modulating general stress responses

DVU3305 may be part of a three-component system that includes a HK, DVU3304, and another RR, DVU3303, that has an unusual domain structure containing a lon protease. DVU3305 regulates its own operon as well as its upstream genes DVU3302-3298, which encode membrane and hypothetical proteins, some of which have UspA type domains (Figure 6; Table S5 in Additional file 1). The two operons have good expression correlation to each other (Figure S3 in Additional file 3). DVU3303-3305 and/or DVU3298-3302 are upregulated during various stresses, such as high pH [35], heat shock [36], and stationary phase [37]. DVU3303 may require phosphorylation to be protease-active, which in turn may be part of the general stress response. The other targets for this RR are the operon DVU2822-2825, which encodes a putative dicarboxylate transporter and pyruvate formate lyase genes, and the high affinity potassium transporter DVU3334-3339 (see below).

#### Potassium uptake genes are controlled by multiple RRs

DVU3334 regulates its own operon, encoding the high affinity potassium uptake genes (*kdpFABC*). The HK in this operon appears to be split into two ORFs, with DVU3336 having the K^+ ^sensor domain and an UspA domain, and DVU3335 having the HK dimerization and phosphoacceptor domain. Interestingly, two other RRs also target the Kdp operon - DVU0596 and DVU3305 - suggesting that potassium uptake is a response to other stresses as well. The putative binding sites for DVU3305 and DVU3334 upstream of the Kdp genes also overlap (Table S9 in Additional file 1).

DVU0596 has a LytR type DBD, and its other targets are two copies of a putative carbon starvation (*cstA*) gene that lie downstream of this RR-HK operon. In *E. coli *the *cstA *gene is upregulated upon exhaustion of carbon source in the medium, and appears to be a peptide permease. Activated DVU0596 may also function by regulating the putative *cstA*. Presumably due to the regulation of the DVU3334-3339 Kdp operon by multiple RRs, the genes in this operon do not demonstrate simple correlation patterns with the co-cistronic RR DVU3334 (Figure S3 in Additional file 3).

#### Regulation of acetyl-CoA levels

A second LytR-type RR, DVU0749, has four genes/operons as its targets (Figure 4). DVU2969 is annotated as an acetyl-CoA synthetase similar to DVU0748, which is in the same operon as the RR itself. DVU2970 has acyl-CoA synthetase and acetyltransferase domains. These seem to suggest that this RR is involved in maintaining acetyl-CoA levels in the cell. Based on the gene annotations for DVU0443-0448, they may constitute a cAMP-dependent membrane transporter or signal transduction protein. Regulon prediction, based on the gene neighbor method [17], also associates this operon with DVU2969. Additionally, DVU0749 has an atypical receiver domain, with the phosphorylatable aspartic acid replaced by a glycine. Since it also lacks a proximally encoded HK, it is likely that this RR is not activated by phosphorylation.

#### Two component system involved in energy metabolism regulation

DVU2394 regulates the DVU2405-2397 operon, which encodes the alcohol dehydrogenase and heterodisulfide reductase genes (Figure 6). The *adh *gene is necessary for growth with ethanol as electron donor, but was also highly expressed during growth on lactate, pyruvate, formate or hydrogen as the electron donor [38]. The operon encoding the RR and DVU2405-2397 are positively correlated, but they appear to be anti-correlated to the other targets DVU3298-3305, which are part of the general stress response (Figure S3 in Additional file 1).

#### Response to nitrite stress

Nitrates and nitrites are known to inhibit sulfate reduction, and thus pose a stress for sulfate-reducing bacteria. DVU0621 targets its upstream genes DVU0624-0625, which encode a nitrite reductase and are highly upregulated in the presence of nitrite [39,40]. The nitrite reductase protein has been crystallized and biochemically shown to reduce nitrite as well as sulfite [41,42]. The tiling array data indicate DVU0624-0625 to be highly expressed in the absence of nitrite (Figure S1 in Additional file 3) [19], and therefore suggest roles in addition to responding to nitrite stress. DVU0621 also targets the lactate/pyruvate oxidation genes DVU3025-3033, and another hypothetical gene, DVU3384.

#### The nitrogen regulator is the most conserved among related species

Despite the identification of a target gene via EMSA, no DAP-chip data could be acquired for RR DVU3220, since conditions for qPCR enrichment of the target could not be found. However, DVU3220, along with DVU1083, are the only two RRs that are conserved in all sequenced *Desulfovibrio *and other related genomes as well (Figure 8). DVU1083 is the phosphate regulator, and it seems likely that DVU3220 is the nitrogen regulator. The identification (by EMSA; Figure 3) of DVU1231-1234, the operon encoding the ammonium transporter, the nitrogen regulatory protein PII, and the P-II uridylyltransferase, as its target supports this hypothesis.

#### Regulation through sRNAs

The RR DVU0679 has as its target a sRNA that lies downstream of the RR gene (Figure 4) that has been annotated as Dv_sRNA2 (Bender, personal correspondance). Tiling array data [19] confirm the expression of this sRNA during normal growth (Figure S1 in Additional file 3), but its function is unknown. The validated binding site motif is present in other unique DAP-chip hits, which are not in upstream regions but may be physiologically relevant. It may be that there are as yet undiscovered non-coding RNAs present near these binding sites, or that binding within coding regions or within an operon presents additional ways of modulating expression of the target genes [43].

### Functional validation using reporter assays

For several of the RRs, the corresponding target promoters were tested using *in vivo *transcriptional reporter analysis. Examination of two component system RRs in *D. vulgaris *would require the knowledge of the activating signals. To bypass this requirement, we sought to examine the binding specificity in the heterologous host *E. coli*. The assay utilized two compatible plasmids: pETDEST42, which expressed the RR from an IPTG-inducible promoter; and pBbS2K-RFP, which expressed the red fluorescent protein (RFP) gene under the control of the target *D. vulgaris *promoter (Materials and methods; Figure S4 in Additional file 3). Expression of RRs in the reporter strains was confirmed using anti-His immunoblots (Figure S4 in Additional file 3).

**Figure 10:**
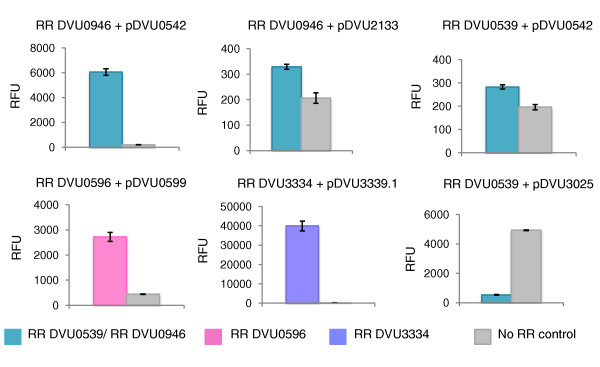
**RFP reporter assay in *E. coli*. Each panel represents activation of a specific promoter by a specific RR as measured in RFUs (relative fluorescence units)**. RFUs were calculated as the ratio of RFP fluorescence to cell growth at OD_590_. Data shown are for 23 hours after start of assay. Error bars represent standard deviation. Grey columns represent negative controls where RFP fluorescence was measured in the absence of any RR (empty pETDEST42 vector). RR, response regulator.

As shown in Figure 10, activation of expression was observed for several RR-promoter combinations. In the case of the two paralogous RRs DVU0946 and DVU0539, both showed transcription from the promoter pDVU0542. Interestingly, RR DVU0539 decreased the background activity of pDVU3025, suggesting that it may act as a transcriptional repressor at this promoter (Figure 10). Not all constructs tested provided meaningful results (Figure S4 in Additional file 3), which could be due to several reasons, such as the absence of a required transcriptional factor, insufficient RR phosphorylation and leaky promoters. However, using the heterologous *E. coli *background did allow us to confirm functional transcription of several target genes by the corresponding RRs.

### Binding site motifs are conserved across related species

The presence of orthologous RRs with conserved binding motifs and target genes in other sequenced microbes is a strong indicator of similar function. Orthologs of *D. vulgaris *RRs could be tracked in several other genomes (Figure 8). We screened 23 sequenced genomes that contained orthologs of any of the *D. vulgaris *Hildenborough RRs for the presence of conserved binding motifs and target genes. These included six *Desulfovibrio *genomes, specifically, the closely related *D. vulgaris *Miyazaki, *D. desulfuricans *G20, *D. salexigens*, the magnetotactic *D. magneticus*, the rumen isolate *D. desulfuricans 27774 *and the human isolate *D. piger*. Two RRs that are conserved in all these species were DVU1083, the phosphate regulator, and DVU3220, a possible nitrogen regulator. DVU3220 is also conserved in some of the other sulfate-reducing bacteria we examined, namely *Desulfomicrobium baculatum, Desulfohalobium retbaense, Desulfobacterium autotrophicum, Desulfatibacillum alkenivorans*, and *Desulfotalea psychrophila *(Figure 8).

DVU3023, the lactate responsive RR in *D. vulgaris*, is conserved in all the environmental *Desulfovibrio *isolates, as well as *D. baculatum, D. retbaense *and *D. psychrophila*, where the binding site motif is also conserved upstream of the central lactate-pyruvate oxidation genes. Similarly, the alcohol dehydrogenase regulator DVU2394 is also conserved in many of the sulfate reducers, although the validated motif for *D. vulgaris *Hildenborough was only found in *D. vulgaris *Miyazaki (Figure 8).

The flagella regulator DVU1063 is conserved among the *Desulfovibrio *spp., being absent in only the non-motile *D. piger*. It is also present in the related pathogen *Lawsonia intracellularis *and in *D. baculatum*, and the binding site motif is also conserved upstream of several flagella and motility related genes in these species (Table S9 in Additional file 1). The pili assembly regulator DVU2114 and its binding site motif upstream of pilin genes are conserved in only a few species - *D. vulgaris *Miyazaki, *D. desulfuricans *G20 and *Syntrophobacter fumaroxidans *(also a sulfate reducer with propionate as electron donor). A DVU2114 ortholog is also present in *D. magneticus*, but this genome lacks the target pilin genes and a similar binding site motif was present upstream of a different gene (Table S9 in Additional file 1).

Orthologs of the paralogous RRs DVU0946 and DVU0539 and their binding site motifs are conserved in all the environmental *Desulfovibrio *spp. as well as other sulfate reducers such as *D. baculatum, D. retbaense *and *D. alkenivorans *(Figure 8). Interestingly, *D. vulgaris *Miyazaki and *D. retbaense *also have paralogous copies for this RR. The orthologs in *D. desulfuricans *G20 and *D. vulgaris *Miyazaki present a case where the motif occurs upstream of additional genes different from those identified in *D. vulgaris *Hildenborough. These genomes have the motif upstream of orthologs of the target DVU0943, but they also have it upstream of a putative split soret cytochrome c precursor gene that is not a target in *D. vulgaris *Hildenborough (Table S9 in Additional file 1). These additional genes may be true targets for the respective RRs, and our genome scans can be a valuable tool for identifying potential targets.

DVU2934, which targets lipid A biosynthesis, has several orthologs, including those found in non-sulfate reducers such as *Geobacter lovleyi *and *Acidobacterium capsulatum*. However, the validated motif was present upstream of the target *lpxC *gene only in *D. vulgaris *Miyazaki and *D. desulfuricans *G20 (Figure 8; Table S9 in Additional file 1). It is possible that the other RRs may have evolved to have different functional roles. Alternately, the RR may have diverged to recognize a different motif upstream of the same target. Suggestive of this latter occurrence, in *S. fumaroxidans*, the *lpxC *gene is directly upstream of the ortholog RR operon, suggesting that its regulation is likely to be linked to the orthologous RR despite the absence of the motif.

Orthologs for the general stress responsive RR DVU3305 are present in some of the *Desulfovibrio *species, *D. baculatum *and *D. retbaense*, and also in less related species such as *Thermodesulfovibrio yellowstonii, Thioalkalivibrio *spp. and *Dechloromonas aromatica*. In most of these genomes, the RR is also associated with the lon-protease RR ortholog of DVU3303, and the binding site is also conserved, indicating that their functions are likely to be related (Figure 8; Table S9 in Additional file 1).

The potassium responsive RR DVU3334 has orthologs only in *D. vulgaris *Miyazaki and *S. fumaroxidans*, where the motif is also conserved (Figure 8). Interestingly, other *Desulfovibrio *species and sulfate reducers do not have the high affinity potassium uptake Kdp genes.

The sRNA regulating RR DVU0679 has orthologs in some of the *Desulfovibrio *species and in another sulfate reducer, *Desulfotomaculum acetoxidans*, and in *Syymbiobacterium thermophilum*. Genome scans show that a target sRNA with a conserved binding site may lie downstream of the RR orthologs in *D. vulgaris *Miyazaki and *D. desulfuricans *G20 (Table S9 in Additional file 1), suggesting that sRNA regulation is a function of these orthologous RRs as well. Genome scans of *D. acetoxidans *and *S. thermophilum *also showed that the highest scoring hits to the motif were not upstream of coding regions (Table S9 in Additional file 1).

Other RRs such as DVU1419 and DVU3381, which target hypothetical genes and therefore have unknown functions, are conserved along with their binding sites in closely related species. Orthologs of RRs without binding site motif predictions, specifically DVU0621, DVU0653, DVU0804, DVU0744, DVU2675, and DVU2577, are limited to a few of the *Desulfovibrio *species (Figure 8).

Conservation of RRs in other Deltaproteobacteria, such as *Geobacter *species and the myxobacteria, are shown in Figure 8. Three RRs do not have any other *Desulfovibrio *orthologs, although orthologs may be present in more distant species. These include DVU1156 and the pDV1 encoded DVUA0137, with no functional predictions from our study, and DVU2588, which may be involved in lactate utilization. The binding site motif for DVU2588 is also conserved in *S. fumaroxidans *and *A. capsulatus *but is present upstream of genes different from those in *D. vulgaris *(Figure 8; Table S9 in Additional file 1).

## Conclusions

Prior to our study, very little was known about the two component regulatory network in sulfate-reducing bacteria. Here we provide a fairly comprehensive map of genes that are transcriptionally regulated by the majority of the two component systems in this model sulfate-reducing organism (Figure 7). Our results include 200 target genes for 24 response regulators and provide strong predictions for the corresponding two component systems that include the regulation of cell motility (flagella and pili), exopolysaccharide production, energy metabolism (lactate utilization, alcohol dehydrogenase regulation, acetyl-CoA levels), lipid A synthesis, nitrogen and phosphate metabolism, and in the responses to stresses such as low potassium, nitrite and carbon starvation. Functions such as lactate utilization and potassium uptake genes are regulated by multiple RRs and appear central to the stress response in *D. vulgaris*. In addition, the experimentally confirmed binding motifs for several of these RRs could also be used to assess gene targets and function of the orthologs of these regulators in related bacteria. With the exception of a few RRs (for example, DVU1083 (PhoB) and DVU2934), most of the DBD-containing RRs encoded in *D. vulgaris *Hildenborough appear to be unique to the sequenced *Desulfovibrio *species and closely related sulfate reducers. This suggests that responses modulated by most two component systems in these sulfate reducing bacteria are unique to the ecological niches they occupy. In addition, the significant numbers of hypothetical proteins and genes with unknown functions amongst the regulated candidates indicate that there remains a lot to be learnt about the environmental stresses faced in these ecosystems. Deeper knowledge of stress response and regulation in these environments is required for robust bioremediation approaches and a better understanding of biogeochemical processes mediated by these bacteria.

## Materials and methods

### Cloning of response regulator genes in *E. coli *

*D. vulgaris *Hildenborough was grown on LS4D medium [44], and the cell pellet was used to purify genomic DNA with the Qiagen Genomic DNA buffer kit, and the Qiagen midi column (tip 100/G). The response regulator gene was PCR amplified and cloned into the entry vector pENTR™/SD/D-TOPO (Life Technologies, Grand Island, NY, USA) with forward primers carrying the 5' sequence CACC. A list of primers used for cloning the genes is in Table S11 in Additional file 1. The entry clone was transformed into Invitrogen's OneShot Top10 chemically competent cells, and selected on LB-Kanamycin plates. Sequencing was used to confirm the presence of the insert. The expression construct was generated by performing an LR recombination reaction (Gateway LR Clonase II, Life Technologies, Grand Island, NY, USA) with the destination vector pETDEST42 (Invitrogen) such that the RR gene is expressed with a carboxy-terminal V5-epitope and a 6X His-tag. The final construct was transformed into *E. coli *BL21 Star (DE3) OneShot chemically competent cells (Invitrogen), and selected on LB-Amp plates. Sequencing was used to confirm the presence of the insert.

### Protein expression and purification

The *E. coli *BL21 (DE3) pETDEST42-RR strains were grown on LB-carbenicillin at 37°C. At mid-log phase, the cells were induced with 0.5 mM IPTG (isopropyl-beta-D-thiogalactopyranoside), and then grown at RT for 24 hours. The cells were pelleted, and resuspended in HisTrapFF wash/binding buffer (40 mM imidazole, 500 mM NaCl, and 20 mM sodium phosphate, pH 7.4). Lysozyme (1 mg/ml) and 1X Novagen's benzonase nuclease were added to the cell suspension. The cells were lysed using a French Press at 4°C, and the cell lysate was clarified by spinning at 10, 000 rpm at 4°C. To check for over-expression, a sample was run on 4 to 12% Bis-Tris gel, the gel was transferred onto a nitrocellulose membrane, and a western blot was performed with mouse anti-6X His-tag antibodies. Prior to purification using the AKTA Explorer, the clarified lysate was filtered through a 0.45 μm syringe filter. The lysate was loaded onto a 1 ml HisTrapFF column (GE Healthcare, Piscataway, NJ, USA) that had been equilibrated with HisTrapFF wash/binding buffer (10 ml). The column was washed with 20 ml of the wash buffer, and then eluted with a linear gradient of 0 to 100% elution buffer (500 mM imidazole, 500 mM NaCl, and 20 mM sodium phosphate, pH 7.4). The pooled protein fractions were stripped of imidazole using a HiPrep 26/10 Desalting column (GE Healthcare) and washing with a desalting buffer (500 mM NaCl, 20 mM sodium phosphate, pH 7.4). The protein preps were concentrated using a high molecular weight cutoff spin filter, and glycerol (50%) and DTT (0.1 mM) were added to the preparations for storage. Examples of purified proteins are shown in Figure S6 in Additional file 3.

### Preparing substrates for EMSAs

Oligonucleotides (oligos; unlabeled and 5'-biotinylated) were ordered from IDT, San Diego, CA, USA. Full length (200 to 400 bp) upstream regions of target genes (Figure 3) were prepared by PCR amplification from *D. vulgaris *Hildenborough genomic DNA using one unlabeled oligo and one 5'-biotin-labeled oligo. A list of oligos is given in Table S2 in Additional file 1. The biotin-labeled substrates were then gel-purified from agarose gels using Qiaquick gel extraction kits (Qiagen Inc, Valencia, CA, USA). Substrates for validating binding site motifs (Figure 9) were prepared by annealing oligos carrying the motif to be tested and approximately 10 bp on either side (Table S6 in Additional file 1). The top strand biotinylated oligo was mixed with a slight excess of unlabeled bottom strand oligo in 10 mM Tris HCl, pH 8.0, 1 mM EDTA, and 50 mM NaCl, and heated to 95°C for 5 minutes followed by slow cooling to 25°C. The annealed substrate was then diluted to 1 pmol/μl.

### Electrophoretic mobility shift assay

EMSAs were performed using the Pierce Lightshift chemiluminescent kit (Thermo Fisher Scientific, Rockford, IL, USA). The response regulator to be tested was mixed with 50 to 100 fmol of biotinylated DNA in 10 mM Tris HCl pH 7.5, 1 mM DTT, 50 mM KCl, 5 mM MgCl_2 _and 25% glycerol. Poly dI-dC (1 μg/ml) was added as a non-specific competitor. The reactions were incubated at 25 to 30°C for 20 minutes, and the reactions were loaded on a pre-cast mini 6% polyacrylamide-0.5X TBE gel (Life Technologies), and run at 100 V at room temperature. The gels were transferred to a nylon membrane by semi-dry blotting (BioRad, Hercules, CA), the nylon membranes were UV cross-linked for 3 minutes, and the blot was developed using the Pierce Chemiluminescent Nucleic Acid Detection kit (Thermo Fisher Scientific). The blot was scanned using the Typhoon 8600 Imager (Molecular Dynamics/Amersham Pharmacia (Piscataway, NJ, USA)).

### Binding reactions with genomic DNA and DNA affinity purification

*D. vulgaris *Hildenborough genomic DNA was prepared using a Qiagen genomic tip and genomic buffer set. The DNA was sheared by sonication to an average length of 500 bp. Binding reactions (100 μl) were set up with 2 to 3 μg of sheared genomic DNA, and the appropriate amount of purified response regulator, in 10 mM Tris HCl, pH 7.5, 1 mM DTT, 5 mM MgCl_2_, 50 mM acetyl phosphate and 25% glycerol. The amount of protein used in the binding reactions was determined based on activity of protein in EMSAs (Table S3 in Additional file 1). Acetyl phosphate was used as a generic method to activate the RRs based on protocols described in the literature [20]. The reactions were incubated at 25°C in a thermal cycler for 30 minutes; 10 μl of the reaction was saved as input DNA. The rest was allowed to bind to 30 μl of Ni-NTA resin that had been washed in the binding/wash buffer (10 mM Tris-HCl, pH 7.5, 5 mM MgCl_2_, 50 mM KCl, 25% glycerol). After 30 minutes of binding, the tubes were spun down to remove the unbound DNA. The resin was washed three times in 100 μl of the wash buffer, and then the bound DNA was eluted with 35 μl of elution buffer (20 mM sodium phosphate buffer pH 8, 500 mM NaCl, and 500 mM imidazole); 35 μl of this elution buffer was also added to the input DNA. The input and the enriched DNA fractions were cleaned up using Qiaquick PCR purification columns (Qiagen Inc).

### Whole genome amplification

The input and enriched DNA samples (10 μl; after clean up) were subjected to whole-genome amplification using Sigma WGA2 kit (Sigma-Aldrich, St. Louis, MO, USA)). Since the starting material was sheared genomic DNA, the first fragmentation heat step was omitted and the number of amplification cycles was increased to 20 as per the manufacturer's suggestions. The amplified samples were cleaned up using Qiagen spin columns and quantified using the nanodrop.

### Quantitative PCR

qPCR was performed on the whole genome amplified input and enriched DNA samples using the PerfectA Sybr Green Mix with ROX (Quanta Biosciences, Gaithursburg, MD, USA). The DNA samples were diluted to a concentration of 2 ng/μl, and 5 μl of each sample was used as the template. The primers used for qPCR of target upstream regions are listed in Table S2 in Additional file 1. Each reaction was done in triplicate. Delta C_T _was calculated as the difference in the C_T _values of the input and enriched samples (ΔC_T _= C_T _(input) - C_T _(enriched). Fold enrichment was calculated as 2^ΔCt^. If the target was found to be at higher amounts in the enriched sample, then the samples were Cy3/Cy5 labeled and hybridized to the chip. If no enrichment of the target was observed, then the DAP-whole-genome amplification steps were repeated under different conditions (usually by varying the protein amount) until enrichment was obtained. For each RR tested, the upstream region of a randomly selected gene was also tested to ensure that non-specific gene targets were not enriched. For most RRs, the negative control used was the upstream region of DVU0013. The exceptions were DVU3234 used for RR DVU1083, DVU0599 used for RR DVU1063, and DVU1083 used for RR DVU0946.

### DNA labeling and hybridization

Cy3- and Cy5-labeled 9-mers were obtained from Trilink Biotechnologies (San Diego, CA, USA). One microgram each of the input and enriched DNA was labeled with Cy3 and Cy5, respectively, using Klenow polymerase (3'-5' exo-, 50, 000 U/ml, New England Biolabs, Ipswich, MA, USA) at 37°C for 2 hours in the dark. The reactions were stopped by adding EDTA to a final concentration of 50 mM. The DNA was precipitated using NaCl and isopropanol, and the pellet was rinsed in 80% ethanol, and air-dried. The pellets were resuspended in 25 μl water, and quantified using the nanodrop. Six micrograms each of the Cy3- and Cy5-labeled DNA were pooled together, and vacuum dried. The pellet was resuspended in 5 μl water, and hybridized overnight at 42°C to a custom *D. vulgaris *Hildenborough 385K tiling array (Roche-Nimblegen, Madison, WI) as per the manufacturer's instructions. The tiling array used 50-mer probes with a 46-bp overlap in the intergenic regions, and a 20-bp overlap in the coding regions. The slides were washed, and scanned on a GenePix 4200A scanner. Each slide was scanned at wavelengths of 532 and 635 nm, and the images were saved separately.

### Data analysis to generate binding site peaks

Nimblescan software v.2.4 (Roche-Nimblegen) was used to grid the 532 and 635 nm images, and to generate the pair files that contain signal intensities for each probe on the array. Scaled log_2_R ratios were then computed for the probes. Nimblescan then used a sliding window of 500 bp to search for peaks that have more than four probes above a cutoff value. The cutoff value is a percentage of the hypothetical maximum log_2_R ratio (mean + 6 standard deviation) that starts at the most stringent value of 90% and decreases in steps of 1 to 15%. The ratio data are randomized 20 times to evaluate the possibility of false positives. As the method to estimate the FDR scores for the peaks, we chose to optimize for strong peaks of varied width. The peak file generated by Nimblescan gives the location of the peak (genomic or plasmid), the start and end loci for the peak, a score that is the log_2_R ratio for the fourth highest probe in the peak, the cutoff percentage (cutoff_p) at which the peak was identified, and the FDR score for the peak. We used MicrobesOnline [16] to map the peak loci to the upstream regions of the gene targets. Nimblescan guidelines state that FDR scores of 0 to 0.05 are indicative of a high-confidence binding site, while peaks with scores of up to 0.2 may also be considered as binding sites. Since our DAP-chip data sets had a large number of peaks with FDR scores less than 0.2, a combination of high log_2_R scores, high cutoff_p values, and low FDR scores was used to identify the most likely targets for each RR. The list of targets presented in Table S5 in Additional file 1 and Figures 4, 5 and 6 is a conservative estimate of the most likely targets. Peaks falling within coding regions, or those that appear in multiple DAP-chip sets, or those with lower scores or higher FDR values were not considered unless they were also found to carry a binding site motif, or appeared to be functionally related to the other top peaks. For DVU1083, where the positive target did not appear in the top hits, DAP-chip was conducted in triplicate and the common hits from the three sets were pooled together to generate a target list. The array data have been deposited in NCBI's Gene Expression Omnibus [GEO:GSE25163] [45].

### Binding site motif predictions for the RRs

MEME was used (through MicrobesOnline [16] and The Meme Suite [46]) to predict motifs in the upstream regions. For each RR, motif predictions were run on the upstream regions of the positive target orthologs, and for those RRs that had more than one target MEME was also run on the upstream regions of the target hits obtained from the DAP-chips. Genomes of interest were scanned for the resulting motif using Perl scripts based on the methods as described in Rhodius *et al*. [47].

### Sigma54 promoter predictions

The *rpoN *matrix was downloaded from Promscan [48]. Promscan generated an *rpoN *matrix based on the σ54 promoter sequences in more than 80 bacterial species [49]. We used the Perl script to determine the σ54 promoters of *D. vulgaris*. Additionally, to test specific upstream regions, we ran the sequence on the Promscan website directly. A list of σ54-promoters that are regulated by RRs is given in Table S10 in Additional file 1.

### Construction of pBbS2K-promoter-RFP plasmids

Promoter regions (300 bp upstream) of selected *D. vulgaris *genes were PCR amplified using primers (Table S12 in Additional file 1) that have a 20-bp overlap with the target vector. The kanamycin-resistant vector pBbS2K-RFP contains the RFP gene under the control of the P*tet *promoter and *tetR *gene. The RFP gene is repressed by TetR, and can be induced using anhydrotetracycline (aTC). The vector pBbS2K-RFP was PCR amplified using promoter-specific primers that have a 20-bp overlap with the promoter of interest (Table S12 in Additional file 1) and the promoters were cloned in by the Gibson method [50] downstream of the P*tet *and upstream of *rfp *such that they replace the native ribosome binding site. The Gibson reaction was used to transform *E. coli *Top10 electrocompetent cells and transformants were selected on LB-Kan-aTC plates and red-white screening was used to pick positive colonies. Sequencing was used to confirm the presence of the promoter insert in the pBbS2K-promoter-RFP constructs.

### RFP reporter assay in *E. coli *

*E. coli *BL21 (DE3) electrocompetent cells were transformed with equal amounts of pBbS2K-promoter-RFP (pSC101 *ori*, Kan^R^) and pETDEST42-RR (pBR322 *ori*, Amp^R^) plasmids (Figure S4 in Additional file 3), and the transformants were selected on LB-Kan-carbenicillin plates. The colonies were adapted to M9 minimal media as described previously [51]. Overnight cultures grown in M9-Kan-Carb were subcultured 1:100 in fresh media and grown at 37°C for 2 hours. The cultures were induced with 50 μM IPTG and 1 ml of each culture (in triplicate) was transferred to a 24-well plate and the plate was incubated with shaking at 25°C in a Tecan Infinite 200 instrument (Tecan, Männedorf, Switzerland) for 24 hours. Cell growth was monitored by measuring absorbance at 590 nm. RFP fluorescence was measured at 535 nm (excitation) and 610 nm (emission). Relative fluorescence units (RFU) were calculated as the ratio of RFP fluorescence to cell growth.

## Abbreviations

bp: base pair; DAP-chip: DNA Affinity Purified-chip; DBD: DNA-binding domain; DTT: dithiothreitol; EMSA: electrophoretic mobility shift assay; FDR: false discovery rate; HK: histidine kinase; ORF: open reading frame; PCR: polymerase chain reaction; PEP-CTERM: Pro-Glu-Pro carboxy-terminal; qPCR: quantitative PCR; RFP: red fluorescent protein; RR: response regulator.

## Authors' contributions

LR designed the experiments, conducted the experiments, conducted the data analysis and wrote the manuscript. AM designed the experiments, conducted the data analysis and wrote the manuscript. EGL conducted the experiments. PSD helped conduct the data analysis. APA helped to write the manuscript. MNP helped conduct the data analysis and helped to write the manuscript. All authors have read and approved the manuscript for publication.

## Supplementary Material

Additional file 1 **Tables S1 to S12**. Table S1: list of *D. vulgaris *response regulators with DNA-binding domains. Table S2: primers used to amplify substrates for EMSAs for identifying positive control target, and primers for qPCR. Table S3: binding conditions for DAP reactions, and fold enrichment of target as determined by qPCR. Table S4: the top 20 peaks obtained by DAP-chip for 27 response regulators. Table S5: DAP-chip gene targets that are regulated by two component systems (from Figures 4, 5 and 6). Table S6: EMSA substrates used to validate the binding site motifs. Table S7: sequences used to build the DAP-chip target-based motif Weblogo images in Figure 8. Table S8: scan of the DAP-chip hit list for other hits having the motif, and scans of the *D. vulgaris *genome for other upstream regions with the motif. Table S9: sequences used to build the ortholog-based motif Weblogo images in Figure 8. Table S10: predicted sigma54-regulated promoters of *D. vulgaris *among the RR gene targets. Table S11: primers used for cloning the response regulator genes into *E. coli*. Table S12: primers used for making RFP reporter constructs. Click here for file

Additional file 2 **Text describing the determination of positive targets by EMSA**. Click here for file

Additional file 3 **Figures S1 to S6**. Figure S1: gene expression of selected genes in *D. vulgaris *obtained by tiling array, and viewed in Artemis [19]. Figure S2: gene-gene correlations for the paralog RRs DVU0946 and DVU0539 and their targets. Figure S3: gene expression correlations based on the microarray expression data available on MicrobesOnline. Figure S4: reporter system in *E. coli *and western blots of RR expression. Figure S5: example of specificity of motif binding by RR. Figure S6: examples of purified response regulators. Click here for file
