# Supplementary material for: Systematic mapping of two component response regulators to gene targets in a model sulfate reducing bacterium
Source: Genome Biol. 2011 Oct 12;12(10):R99. doi: 10.1186/gb-2011-12-10-r99 (PMC3333781; doi:10.1186/gb-2011-12-10-r99)
Supplement: Additional file 2 — Text describing the determination of positive targets by EMSA. [file gb-2011-12-10-r99-S2.DOCX]

**Additional File 2**

**Determination of positive target by EMSA**. Targets for the following RRs were identified based on a combination of factors as described (Figure 3C). For DVU2934, the DVU2917 locus was tested because it was the nearest gene with a predicted sigma54 dependent promoter and confirmed to be a target. For DVUA0057, the DVUA0089 locus was tested as a target based on published literature that hypothesized DVUA0057 to regulate PEP-CTERM proteins such as DVUA0089 (Haft et al, 2006), and because DVUA0089 also had a predicted sigma54 promoter. For RR DVU0110, a number of proximal genes had predicted sigma54 promoters and were tested but only the upstream region of DVU0132 was shifted. For RR DVU1063, DVU1032 was selected as a candidate not only because it was the most proximal sigma54 regulated gene, but also because of the distribution of orthologs of the RR and the target were conserved in several other sequenced *Desulfovibrio*. For the RR DVU3220 the DVU1231 locus was tested based on functional prediction. DVU3220 is the most conserved of all the RRs among the *Desulfovibrio,* being present in all the sequenced species to date (Figure 8), and can be speculated to be the nitrogen regulator, while DVU1231 encodes a putative ammonium transporter. The RR DVU1156 is unique to *D. vulgaris* Hildenborough (Figure 8), and was successfully tested for binding to a sigma54 regulated target, the DVU1164 locus, which is also unique to *D. vulgaris* Hildenborough.
