# Supplementary material for: Systematic mapping of two component response regulators to gene targets in a model sulfate reducing bacterium
Source: Genome Biol. 2011 Oct 12;12(10):R99. doi: 10.1186/gb-2011-12-10-r99 (PMC3333781; doi:10.1186/gb-2011-12-10-r99)
Supplement: Additional file 3 — Figures S1 to S6. Figure S1: gene expression of selected genes in D. vulgaris obtained by tiling array, and viewed in Artemis [19]. Figure S2: gene-gene correlations for the paralog RRs DVU0946 and DVU0539 and their targets. Figure S3: gene expression correlations based on the microarray expression data available on MicrobesOnline. Figure S4: reporter system in E. coli and western blots of RR expression. Figure S5: example of specificity of motif binding by RR. Figure S6: examples of purified response regulators. [file gb-2011-12-10-r99-S3.DOC]

**Additional File 3**

**Figures S1-S6**

**Figure S1**. **Gene expression of selected genes in *D. vulgaris* obtained by tiling array, and viewed in Artemis**. The red trace denotes the plus strand and green trace denotes the minus strand. The axis is log_2_(normalized intensity
relative to the median probe). **a**. high expression of DVU2917 (*lpxC*); **b**. DVU0621-22, and high expression of DVU0624-0625 (nitrite reductase); **c**. basal expression of DVU0539-0540, and operon DVU0541-0545; **d**. medium expression of DVU0943-0946; **e**. High expression of lactate utilization genes DVU3025-33; **f**. no expression of DVU3284 lactate permease; **g**. high expression of DVU2451 lactate permease; **h**. tiling array shows presence of a small orf/sRNA in between DVU3282 and DVU3283; **i**. sRNA downstream of DVU0679; **j, k**. are there small sRNAs expressed between DVU3136-3137 and DVU0652-0653? All data are available in Price *et al* 2011 [19].

**Figure S2.** Gene-gene correlations for the paralog RRs DVU0946 and DVU0539 and their targets. The microarray expression data available for *D. vulgaris* from Microbes Online was used to generate the correlations chart for the operons DVU0943-946, DVU0539-540, DVU0542-545, DVU2132-2133, DVU2451, and DVU3025-3033.


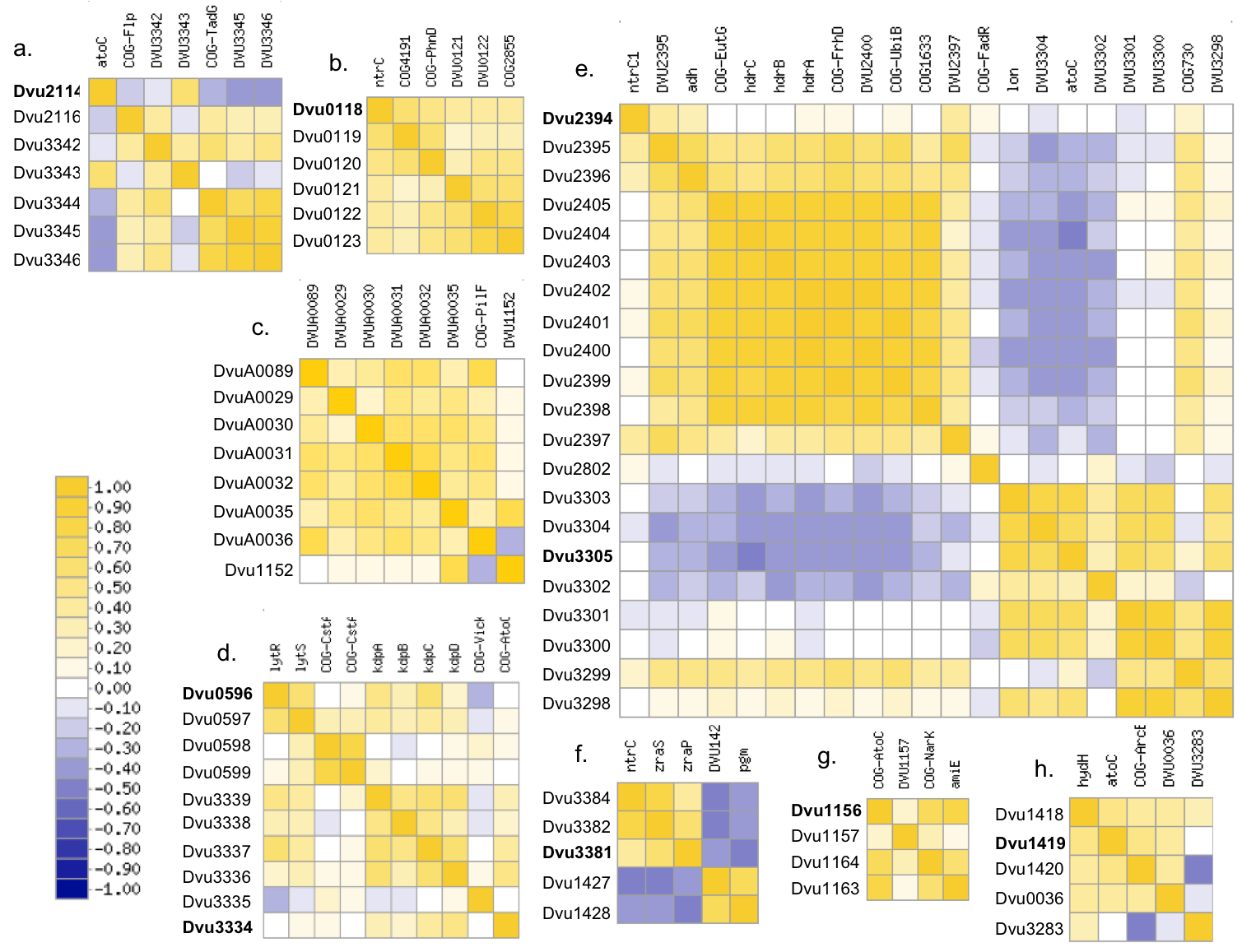


**Figure S3.** Gene expression correlations based on the microarray expression data available on Microbes Online for: a. RR DVU2114 and its target genes; b. RR DVU0118 operon genes and its gene ; c. RR DVUA0057 targets; d. RR DVU0596 and its operon and its targets that include RR DVU3334 and its operon; e. RR DVU2394, its operon, and its targets that include RR DVU3305 and its operon; f. RR DVU3381 and its targets; g. DVU1156 and its targets; h. DVU1419 operon and its targets. RR genes are shown in bold.


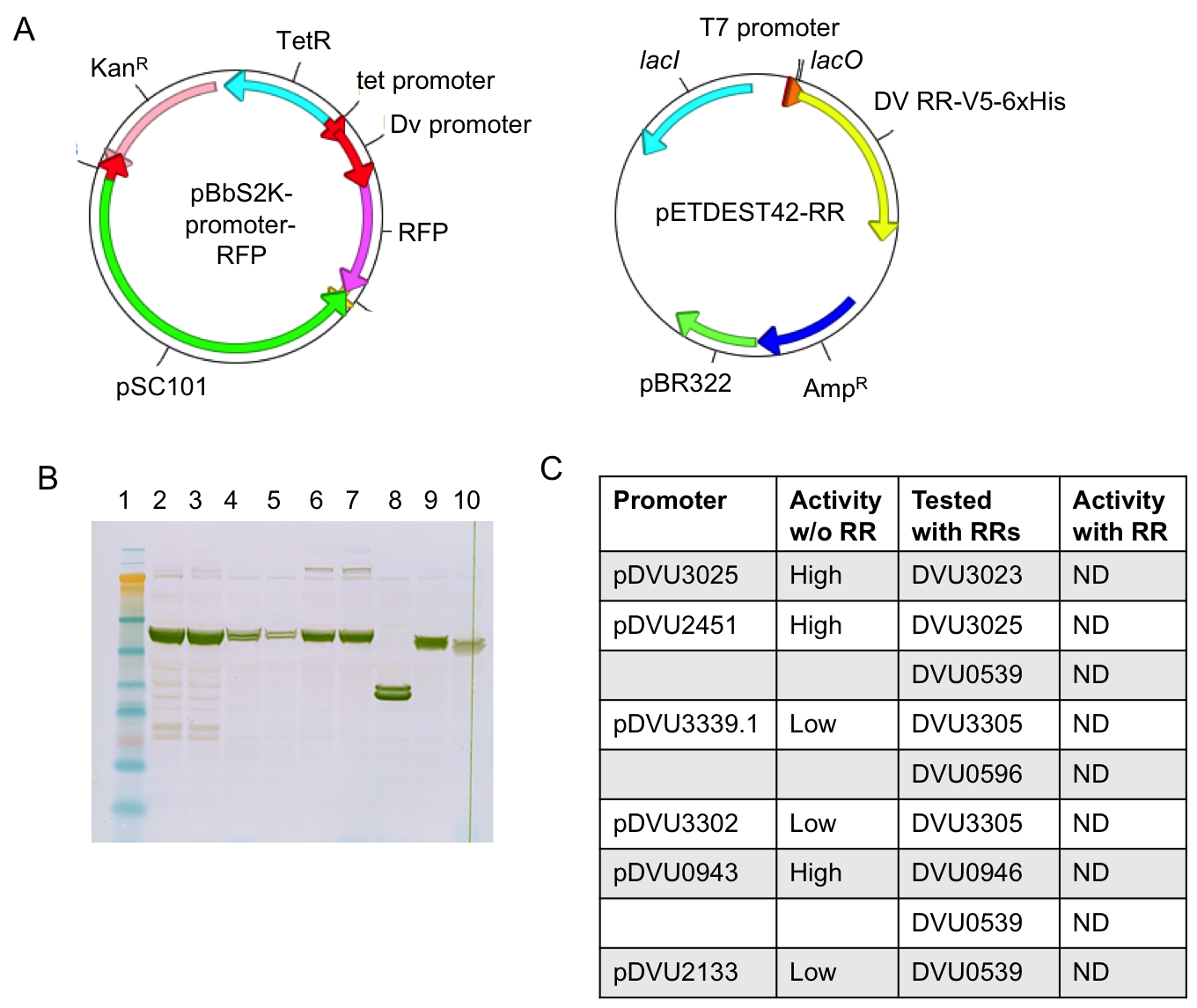


**Figure S4**. Reporter assay in *E. coli*. **A.** The two plasmid transcriptional reporter system in *E. coli*. The pETDEST42-RR plasmid (pBR322 origin, Amp^R^) expresses the *D. vulgaris* RR from a T7 promoter. The pBbS2K-promoter-RFP plasmid (pSC101 origin, Kan^R^) expresses the RFP gene from the *D. vulgaris* promoter. **B.** Western blots showing expression of RR from the reporter strains in cultures induced with 50 μM IPTG. Cultures were grown in M9 minimal media with carbenicillin and kanamycin at 37°C overnight, subcultured 1:100 in fresh media and grown at 37°C for 2.5 hours, induced with IPTG, and grown overnight at RT. Cells were pelleted from 1 ml culture, and whole cell extracts were made by boiling in 1X SDS loading buffer. Supernatants were loaded on a 4-12% Bis-Tris pre-cast gel (Novex, Invitrogen), and run in MES buffer. The gel was transferred to a PVDF membrane, and proteins were detected using anti-His antibodies. Lane 1-marker, lane 2-10 - extracts from reporter strains with the following RR-promoter combinations: lane 2 – RR DVU3023/pDVU3025, lane 3 – RR DVU3023/pDVU2451, lane 4 – RR DVU0946/pDVU0943, lane 5 – RR DVU0946/pDVU0542, lane 6 – RR DVU0539/pDVU0542, lane 7 – RR DVU0539/pDVU3025, lane 8 – RR DVU0596/pDVU0599, lane 9 – DVU3305/pDVU3302, lane 10 – RR DVU3334/pDVU3339.1. **C.** Reporter strains that were tested but did not give meaningful results. High activity without RR indicates background leaky activity from promoter in the presence of an empty pETDEST42 vector. Activity with specific RRs were not detected (ND) above the background for the constructs shown.

**
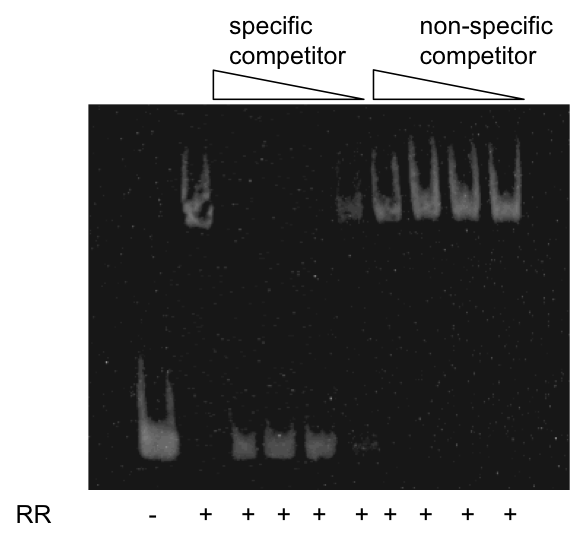
**

**Figure S5 – Example of specificity of motif binding by RR.** RR DVU2934 binds to a DNA sequence motif that is conserved in only very closely related species although the RR itself has orthologs in several species. In order to confirm the specificity of the binding site motif that we discovered, EMSAs with specific and nonspecific competitors were performed. Binding reactions were set up with 10 μl purified DV2934 RR and 100 fmol of biotin-labeled wild-type motif, and unlabeled wild type motif at 100, 50, 25, 5 pmol was added as the specific competitor, and a modified motif at 100, 50, 25, 5 pmols was added as the non-specific competitor.

**Figure S6.**  Examples of purified response regulators. RRs were run on a 4-12% Bis-Tris gel (Invitrogen) in MES buffer, and Coomassie stained. Left panel shows RRs of the OmpR, LytR, and NarL families (15 μl RR), and right panel shows some of the sigma54-dependent RRs (20 μl). Numbers on the top indicate the RR DVU#, and numbers on the right are the molecular weights of the protein standards (Seeblue Plus2 prestained ladder, Invitrogen).
